# Diagnosis of Lissencephaly in a Neonate After Antenatal Polyhydramnios and Suspicion of Fetal Esophageal Atresia: A Case Report

**DOI:** 10.7759/cureus.92565

**Published:** 2025-09-17

**Authors:** Hirofumi Watanabe, Kyosuke Ibi, Kota Yoneda, Satsuki Kakiuchi

**Affiliations:** 1 Department of Neonatology, Tokyo Women’s Medical University Hospital, Tokyo, JPN

**Keywords:** classical lissencephaly, epilepsy in children, esophageal atresia, fetal diagnosis, magnetic resonance imaging, obstetrics ultrasonography, pregnancy counseling, radiological fetal diagnosis

## Abstract

Polyhydramnios is a non-specific prenatal finding associated with several fetal anomalies, most notably gastrointestinal obstructions, such as esophageal atresia (EA). However, its etiology may extend beyond gastrointestinal causes to include central nervous system (CNS) abnormalities. We report the case of a neonate initially suspected *in utero* to have EA based on polyhydramnios and a small fetal gastric bubble, but postnatally diagnosed with classical lissencephaly associated with Miller-Dieker syndrome. A 32-year-old primigravida was referred to our center at 28 weeks of gestation on account of suspected fetal EA with polyhydramnios and a small gastric bubble on prenatal ultrasonography (US). No other anomaly was identified, and she was admitted for management at 30 4/7 weeks of gestation. The patient was delivered at 36 3/7 weeks of gestation via emergency cesarean section due to premature rupture of membranes and arrest of vaginal labor. The neonate showed no sign of EA, and that diagnosis was excluded based on successful passage of an orogastric tube and normal abdominal radiography. Mild dysmorphic features, such as micrognathia and hypertelorism, were noted. As the etiology of polyhydramnios remained unclear, neuroimaging was performed. Cranial US findings were unremarkable; however, head magnetic resonance imaging (MRI) on day seven of life revealed a thickened cortex lacking normal sulcation, which is consistent with classical lissencephaly. Chromosomal microarray analysis revealed a deletion on 17p13.3, confirming Miller-Dieker syndrome. The patient was discharged on day 35 after an uneventful neonatal period; however, epileptic spasms and developmental delay were noticed from the age of six months. This case highlights the diagnostic challenge of differentiating between gastrointestinal and neurological causes of polyhydramnios. Although EA is a common differential diagnosis in such cases, the potential for CNS malformations mimicking gastrointestinal pathologies must be considered. Routine fetal ultrasound alone may fail to detect subtle cerebral abnormalities such as lissencephaly, especially in the absence of ventriculomegaly or other signs. Fetal MRI, which has superior soft tissue resolution, should be considered when the etiology of polyhydramnios is uncertain, even if there is no overt CNS anomaly. This case highlights the importance of prompt neuroimaging, including fetal MRI, which may facilitate diagnosis and counseling of families, and optimize postnatal care.

## Introduction

Polyhydramnios is an abnormal increase in amniotic fluid volume that is typically diagnosed in the second or third trimester and reportedly complicates 1-2% of singleton pregnancies [1,2]. It is a non-specific finding associated with various fetal conditions, including gastrointestinal obstruction, neuromuscular disorders, and central nervous system (CNS) anomalies. When polyhydramnios is associated with a small fetal stomach, fetal esophageal atresia (EA) is strongly suspected [3].

Lissencephaly is a cortical malformation caused by neuronal migration disorders. Classical lissencephaly, a narrowly defined form of lissencephaly, is characterized by agyria or pachygyria [4]. Prenatal diagnosis of classical lissencephaly is challenging, and most patients are diagnosed with it in infancy when they develop symptoms such as seizures, feeding difficulty, and developmental delay [5]. Early diagnosis of lissencephaly is beneficial for allowing time for family counseling and establishing a follow-up system. In this report, we present the case of a patient who was postnatally diagnosed with classical lissencephaly, although fetal EA was suspected prenatally because of polyhydramnios and a small fetal gastric bubble. This case illustrates a pitfall in prenatal diagnosis related to polyhydramnios and emphasizes the importance of prompt workup with fetal magnetic resonance imaging (MRI) and consideration of CNS malformations in the differential diagnosis of such cases.

## Case presentation

**Figure 1:**
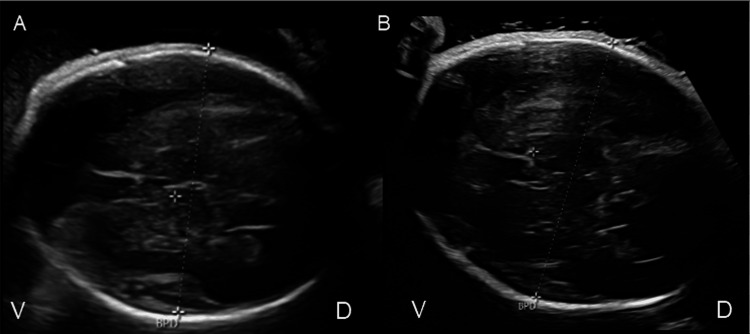
Prenatal ultrasound at (A) 30 and (B) 35 weeks of gestation V: ventral, D: dorsal, BPD: biparietal diameter. Prenatal ultrasound at (A) 30 and (B) 35 weeks of gestation showed no structural abnormality of the fetal brain. Probe marker is directed toward maternal right.

A 32-year-old primiparous Japanese woman with a singleton pregnancy was referred to our hospital at 28 weeks of gestation due to suspected fetal EA after polyhydramnios and a small fetal gastric bubble were detected during routine prenatal ultrasonography (US) at 25 weeks of gestation. She and her husband were non-consanguineous and had no remarkable personal or family history, including that of neurological diseases. She had a history of one spontaneous abortion and was not taking any medication or supplement. The index pregnancy was achieved through in vitro fertilization embryo transfer. On obstetric evaluation at our outpatient clinic, polyhydramnios and a small fetal gastric bubble were noted, but there was no pouch sign. Fetal US detected no other abnormality, and fetal growth remained within the normal range. The patient did not have gestational diabetes mellitus. The same diagnosis of fetal EA was made in our hospital based on the US findings. She was admitted to our hospital at 30 4/7 weeks of gestation for the management of polyhydramnios. No intracranial structural abnormality was identified on repeat fetal US (Figure 1).

**Figure 2:**
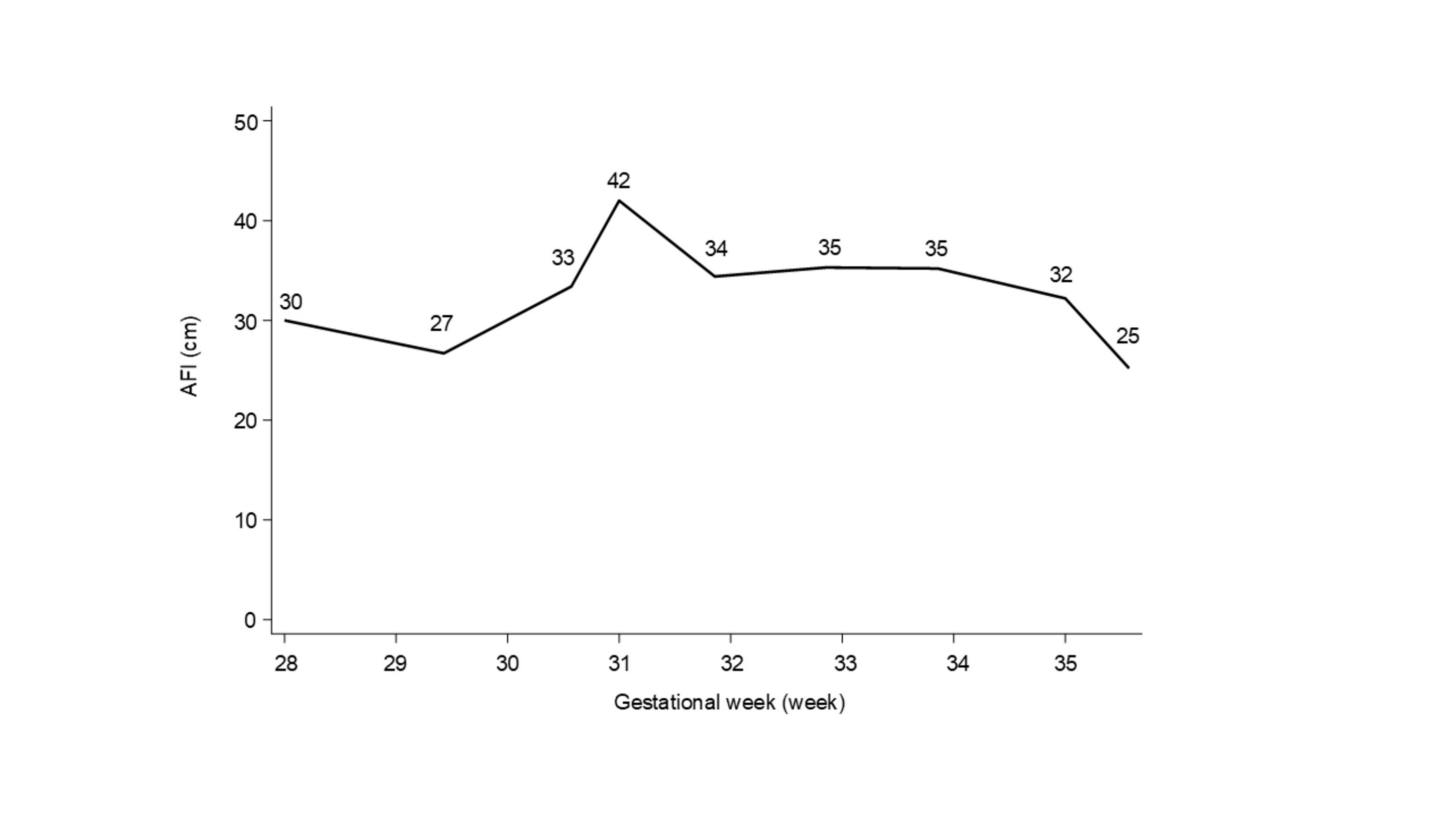
Changes in the amniotic fluid volume after the detection of polyhydramnios at 28 weeks of gestation AFI: amniotic fluid index. The AFI showed a tendency to improve with advancing gestational weeks.

Because no additional abnormalities were detected, further imaging, such as fetal MRI, was not performed. Likewise, no genetic testing, including amniocentesis, was undertaken. The maximum amniotic fluid index (AFI) was approximately 42 cm at 30 weeks of gestation, and therapeutic amnioreduction was deemed unnecessary (Figure 2).

**Figure 3:**
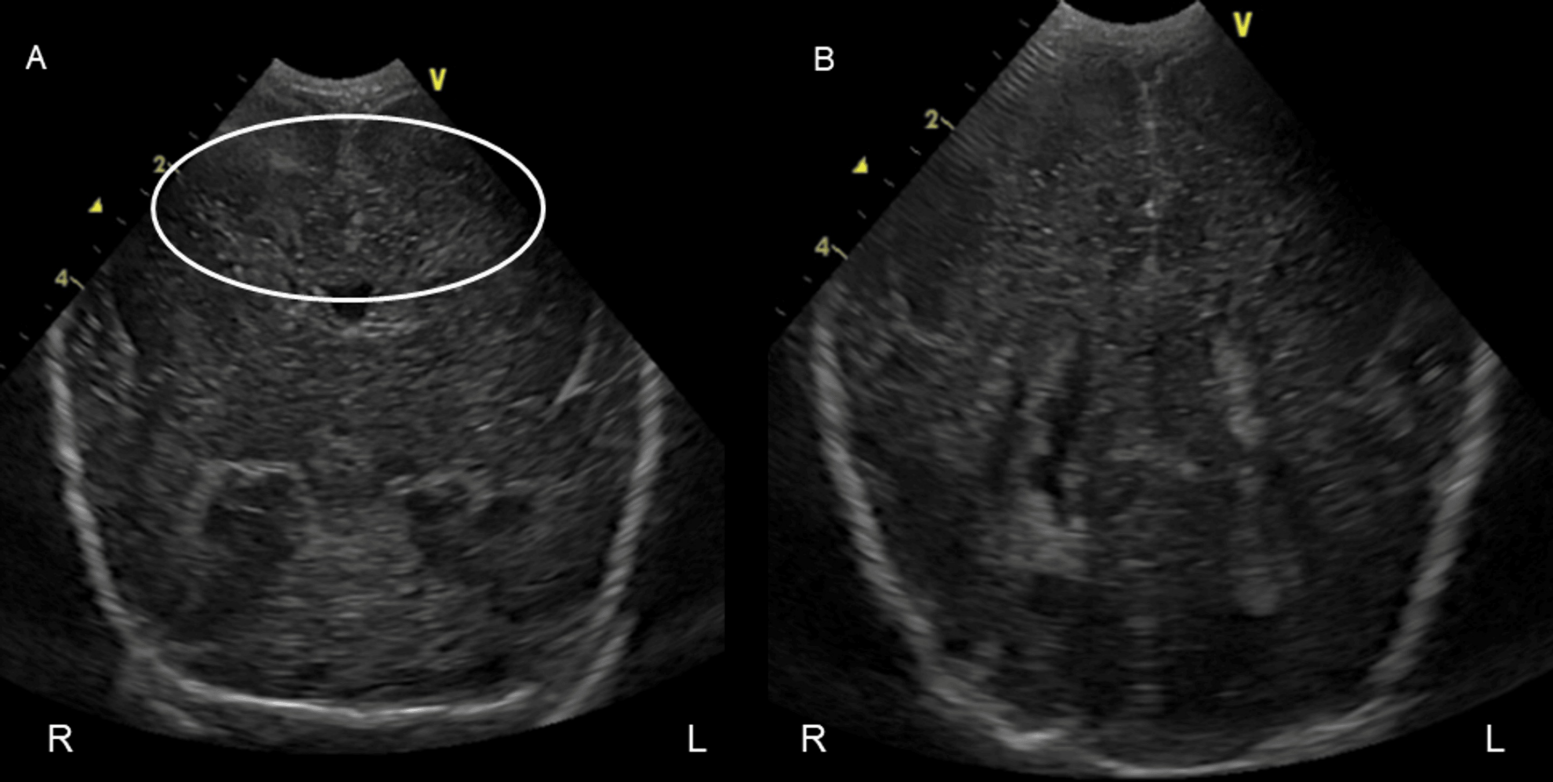
Coronal ultrasonography on the day zero of life R: right, L: left. Retrospective assessment indicated the absence of sulci corresponding to the cingulate gyrus (indicated by the white circle in A).

The neonate was delivered via an emergency cesarean section at 36 3/7 weeks of gestation because of premature rupture of membranes and arrest of vaginal labor. The patient was a male with Apgar scores of eight and nine at one and five minutes, respectively. His length was 45.0 cm (-0.71 standard deviation; SD), weight was 2,059 g (-1.47 SD), and head circumference was 30.3 cm (-1.35 SD). He exhibited no sign of EA, such as excessive frothy salivation, and an orogastric tube was smoothly inserted into his stomach; its position was confirmed with plain chest and abdominal radiography. These findings effectively ruled out EA. Physical examination revealed a slight ocular hypertelorism and micrognathia, but no other external malformation or neurological impairment was detected. Routine blood test results were within normal limits. Cranial US performed at birth did not reveal any apparent structural abnormality in the brain (Figure 3).

**Figure 4:**
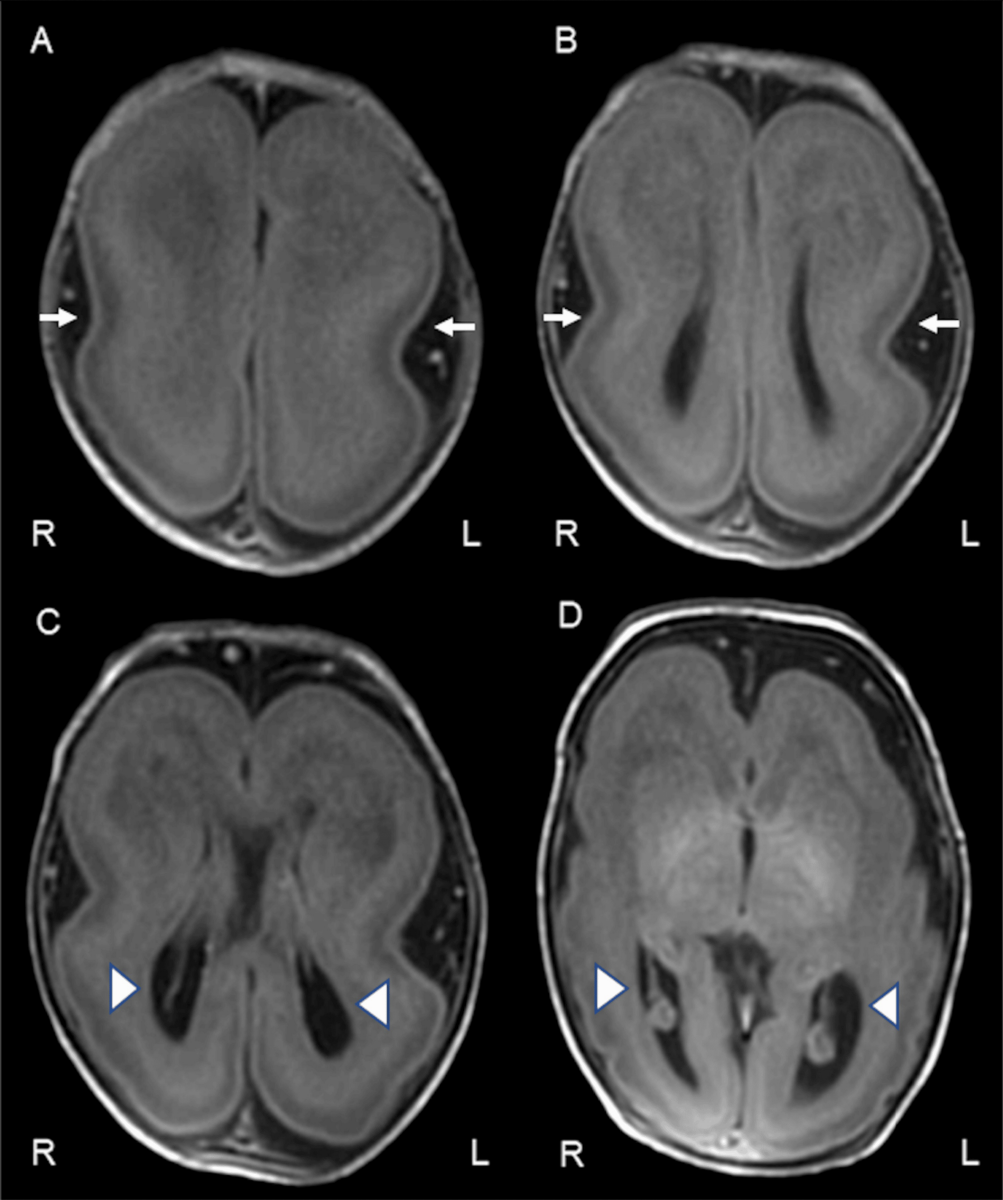
Axial T1-weighted magnetic resonance imaging on day seven of life R: right, L: left. Axial T1-weighted MRI scans on day seven of life demonstrating characteristic features of classical lissencephaly. (A–D) Sequential axial slices from superior to inferior. The images show a diffusely thickened cerebral cortex and absence of normal gyration (agyria), resulting in a smooth brain surface. The “figure-of-8” appearance is notable in images (B) and (C), reflecting shallow Sylvian fissures and simplified sulcation. The lateral ventricles are enlarged and dysmorphic, particularly evident in (C) and (D). White arrows indicate areas of agyria and thickened cortex. Arrowheads mark dysplastic and enlarged lateral ventricles.

Cardiac and abdominal US scans revealed no anomaly. However, given the combination of unexplained polyhydramnios, borderline microcephaly, and dysmorphic facial features, we further evaluated the patient using a brain MRI on day seven of life, which revealed a thickened cortex without gyri or sulci, leading to the diagnosis of classical lissencephaly (Figure 4).

No congenital or acquired infectious disease was detected. Electroencephalography performed on day eight of life did not reveal any epileptiform discharge. Genetic counseling was provided to the parents who expressed a desire to investigate the underlying cause. After obtaining informed consent, G-banding and chromosomal microarray analyses were performed on day 14 of life. A deletion on chromosome 17p13.3. was identified, confirming the diagnosis of lissencephaly associated with Miller-Dieker syndrome. During hospitalization, a slight decrease in muscle tone was noted, but the infant continued to feed well without any clinical symptom, including seizures. He was discharged home on day 35 of life. During follow-up, he developed epileptic spasms at six months of age, and electroencephalography showed multifocal epileptiform discharges. He was diagnosed with infantile epileptic encephalopathy syndrome and commenced on antiepileptic medication. At the latest follow-up at nine months of age, the patient exhibited decreased muscle tone and developmental delay with insufficient eye tracking and head control. He was being fed orally, with some degree of difficulty.

## Discussion

This case presents an intricate diagnostic course in which prenatal findings of polyhydramnios and a small gastric bubble initially led to the suspicion of EA. However, postnatal assessment revealed classical lissencephaly associated with Miller-Dieker syndrome. The clinical importance of this case lies in the potential for CNS anomalies to be associated with features that mimic gastrointestinal tract obstruction on prenatal imaging.

EA is a common cause of polyhydramnios, and other associated prenatal findings may include a small or absent gastric bubble and the presence of the pouch sign [6]. Accurate prenatal diagnosis of EA remains a clinical challenge. US alone is insufficient for diagnosing EA prenatally and has a high rate of false-positive diagnoses. When performed following suspicious US findings, fetal MRI has a good overall diagnostic accuracy for EA [7]. MRI allows detailed visualization of fetal anatomy regardless of fetal position, amniotic fluid volume, or maternal habitus. Increased prenatal detection of EA prompts careful assessment of associated anomalies and provides an opportunity for effective prenatal counseling [8]. Fetal MRI is also useful for detecting abnormalities outside the gastrointestinal tract, such as CNS abnormalities [9]. In the present case, fetal MRI was not performed because no associated findings suggestive of underlying conditions other than fetal EA were identified on fetal US. This decision was partly influenced by anchoring bias, as the patient had been referred to our center with a preliminary diagnosis of EA, which directed the clinical focus away from CNS anomalies. However, retrospectively, fetal MRI may have been useful in this case.

As in the present case, prenatal diagnosis of lissencephaly with routine fetal US screening is extremely challenging. Abnormalities in cerebral sulcation are typically observed at around 26 weeks of gestation, whereas ventricular enlargement tends to appear between 28 and 30 weeks of gestation [10,11]. Typical morphological features usually appear after the recommended time for routine fetal anatomical sonographic scan [12]. Prenatal diagnosis of lissencephaly with fetal US has only been reported in a few cases, typically accompanied by findings such as increased nuchal translucency and ventriculomegaly [13-15]. Further neuroimaging studies including brain MRI should be promptly considered to detect lissencephaly in these patients with slight findings. In the present case, fetal US screening showed no such findings, even after a retrospective evaluation. If brain MRI were taken at around peak AFI in the patient, lissencephaly might be confirmed prenatally, which enable detailed prenatal counseling for the parents. Even during postnatal evaluation, identifying lissencephaly based solely on ultrasound imaging was highly difficult in this case. The absence of sulci corresponding to the cingulate gyrus, a finding suggestive of lissencephaly, was suspected during retrospective review of cranial US images obtained at birth (Figure 2A) [16]. When the etiology of polyhydramnios is not confirmed, neuroimaging studies may be useful diagnostic tools for identifying underlying CNS abnormalities.

This case emphasizes the importance of considering CNS anomalies in fetuses presenting with polyhydramnios and ambiguous gastrointestinal findings. When EA is suspected prenatally but not definitively confirmed, fetal MRI may be indicated not only to clarify gastrointestinal anatomy but also to evaluate brain development.

## Conclusions

Polyhydramnios without a confirmed etiology in the prenatal period warrants comprehensive evaluation, including consideration of CNS anomalies such as lissencephaly. Although EA is a common differential diagnosis, its imaging features overlap with those of CNS disorders, thus necessitating a broader diagnostic approach. This case illustrates how postnatal brain MRI led to the diagnosis of classical lissencephaly in a neonate prenatally suspected to have EA. Fetal MRI may be valuable for distinguishing among the potential etiologies of polyhydramnios, enabled detailed prenatal counseling for the parents. Its utility extends beyond gastrointestinal anomalies to include the detection of subtle cerebral malformations.
